# Supplementary material for: Long-Term Outcomes and Prognostic Factors of Endoscopic Submucosal Dissection for Early Gastric Cancer in Patients Aged ≥75 Years
Source: Cancers (Basel). 2020 Oct 31;12(11):3222. doi: 10.3390/cancers12113222 (PMC7692251; doi:10.3390/cancers12113222)
Supplement: Supplementary file 1 [file cancers-12-03222-s001.pdf]

Supplementary Materials: Long-Term Outcomes and Prognostic Factors of Endoscopic Submucosal Dissection for Early Gastric Cancer in Patients Aged ≥75 Years

Jin Won Chang, Da Hyun Jung, Jun Chul Park, Sung Kwan Shin, Sang Kil Lee and Yong Chan Lee

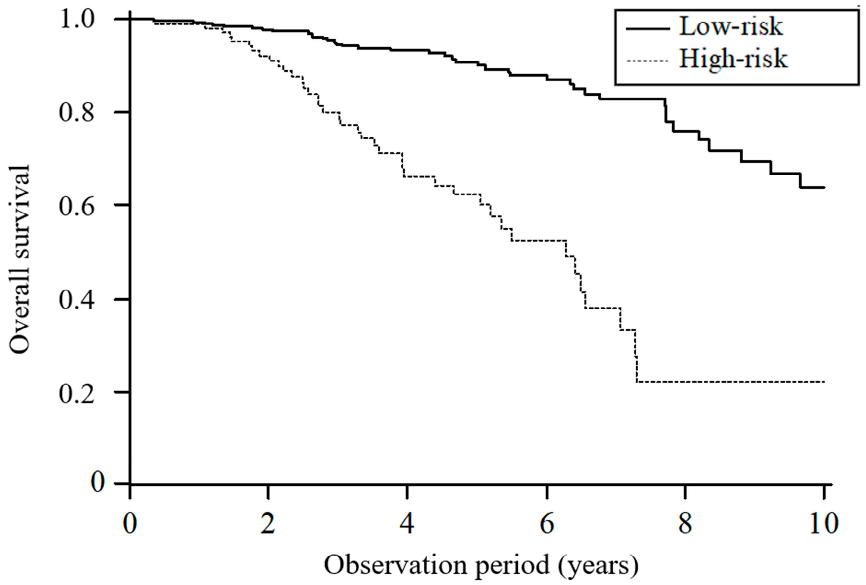

Figure S1. Kaplan–Meier estimation of overall survival (OS) for each group. The 3-, 5-, and 10-year OS rates in the curative resection group were 91.0%, 86.9%, and 58.3%, respectively and those in the non-curative resection group were 91.6%, 72.7%, and 43.8%, respectively. ESD, endoscopic submucosal dissection; EGC, early gastric cancer.

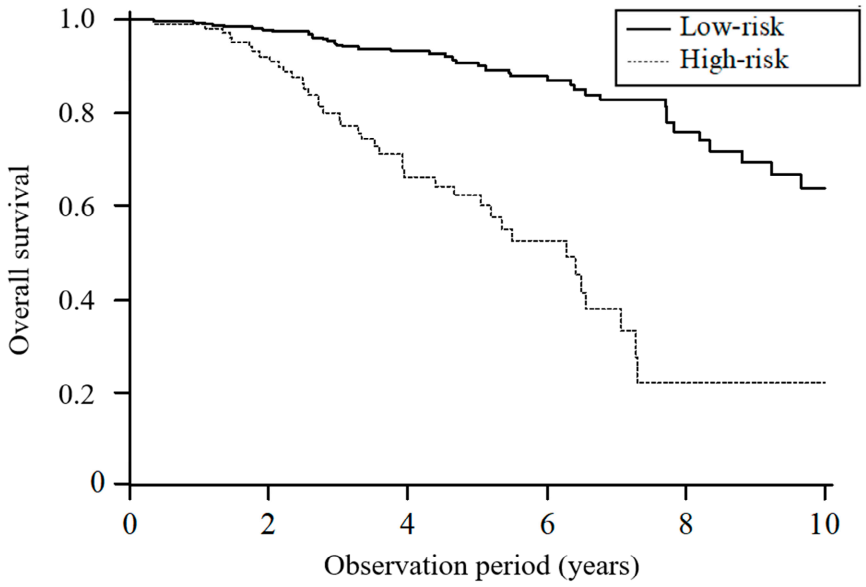

Figure S2. Kaplan–Meier estimation of overall survival (OS) for each group. The 3-, 5-, and 10-year OS rates in the low-risk group were 94.6%, 90.1%, and 63.8%, respectively and those in the high-risk group were 80.0%, 62.5%, and 22.1%, respectively. ESD, endoscopic submucosal dissection; EGC, early gastric cancer.

Table S1. Details of patients who died because of gastric cancer after ESD.

**Patient**	**Pathologic results of ESD**					**Interval between ESD and residual/local recurrence (months)**	**Metastasis**	**Management for residual/local recurrence and metastasis**
	**Tumor size, mm**	**Histologic type**	**Depth of invasion**	**LVI**	**Cut margin**			
1 (78-year-old man)	16	Tubular adenocarcinoma, well differentiated	Submucosa (2700μm)	-	+ (LM)	55.7	Peritoneum	No treatment
2 (76-year-old man)	8	Tubular adenocarcinoma, poorly differentiated	Muscularis mucosa	-	-	87.3	Bone	Palliative chemotherapy
3 (76-year-old man)	10	Tubular adenocarcinoma, well differentiated	Lamina propria	-	-	33.3	Peritoneum, liver	No treatment

ESD, endoscopic submucosal dissection; LVI, lymphovascular involvement; LM, lateral margin

© 2020 by the authors. Licensee MDPI, Basel, Switzerland. This article is an open access article distributed under the terms and conditions of the Creative Commons Attribution (CC BY) license (http://creativecommons.org/licenses/by/4.0/).
